# What are the Aboriginal worldviews of disability in the Fitzroy Valley? Aboriginal Participatory Action Research to develop strategies for decolonising disability services

**DOI:** 10.1136/bmjopen-2024-093608

**Published:** 2025-09-01

**Authors:** Thomas Stubbs, Mudge Bedford, Emma Bear, Emily Carter, Anita Pickard, Jadnah Davies, Sue Thomas, Alexandra L C Martiniuk, Elizabeth J Elliott, Lauren J Rice

**Affiliations:** 1 The University of Sydney, Faculty of Medicine and Health, School of Public Health, Sydney, New South Wales, Australia; 2 The University of Sydney, Faculty of Medicine and Health, Speciality of Child and Adolescent Health, Sydney, New South Wales, Australia; 3 Marninwarntikura Women’s Resource Centre, Marulu Team, Fitzroy Crossing, Western Australia, Australia; 4 Marra Worra Worra Aboriginal Corporation, NDIS Remote Community Connector Team, Fitzroy Crossing, Western Australia, Australia; 5 The University of Toronto, Dalla Lana School of Public Health, Toronto, Ontario, Canada; 6 Westmead, Sydney Children's Hospital Network, Kids Research, Sydney, New South Wales, Australia

**Keywords:** Australian Aboriginal and Torres Strait Islander Peoples, Disabled Persons, Health Services

## Abstract

**Abstract:**

**Objectives:**

Aboriginal and Torres Strait Islander people living with disability have unequal access to health and disability support services. The impacts of colonialism and the deficit-based, Western medical model of disability have been identified as barriers to services in remote Aboriginal communities. This study explored different perceptions of disability and identified strategies to help bridge the gap between Aboriginal community members in the Fitzroy Valley and Western health and disability support services.

**Design:**

Aboriginal Participatory Action Research approach with in-depth interviews. Transcripts were analysed using reflexive thematic analysis. Preliminary results were presented to community representatives for contextualisation, validation and to co-design recommendations.

**Setting:**

Fitzroy Valley in the Kimberley region, Western Australia.

**Participants:**

Aboriginal community members with lived experience of disability (n=7) and health and disability support service providers (n=12).

**Results:**

Eight themes were identified: (1) Aboriginal kinship systems are a community strength and support for people living with disability; (2) Aboriginal people from the Fitzroy Valley perceive disability as a social construct; (3) Western medical model of disability differs from Aboriginal perceptions of disability; (4) Aboriginal people from the Fitzroy Valley perceive different types of disabilities in various ways; (5) good awareness of fetal alcohol spectrum disorder in the Fitzroy Valley, but more education is wanted; (6) focus on functional needs and supports for disability; (7) barriers to disability services and (8) decolonise disability services. Community co-designed recommendations focus on centring the Aboriginal worldviews of disability in the Fitzroy Valley.

**Conclusions:**

Decolonising disability services is needed to improve access for Aboriginal and Torres Strait Islander communities. This should involve adapting the current Western medical model of services to enable strengths-based diagnostic and support services that align with Aboriginal and Torres Strait Islander kinship systems, cultures and ways of being. Community leadership must play a central role in this shift.

**STRENGTHS AND LIMITATIONS OF THIS STUDY**

- Aboriginal community leadership in the design and implementation of the research.
- Gathered in-depth insights into how Aboriginal communities conceptualise disability and access support services.
- Aboriginal community members co-designed recommendations to address the results.
- The findings are not generalisable to all Aboriginal communities, but provide useful insights for policy and further research.

## Introduction

 Aboriginal and Torres Strait Islander people are the world’s oldest continuous civilisation, with an ancestry line dating 75 000 years.^1^ Culture and connection to family, community and lands are fundamental to Aboriginal and Torres Strait Islander health and well-being,^2^ and are a source of strength and resilience.^3^ Colonisation, displacement, disempowerment, trauma and racism contribute to unequal health, economic and social and emotional well-being (SEWB) outcomes between Aboriginal and Torres Strait Islander and non-Indigenous Australians.^4^ Inequalities are exacerbated among Aboriginal and Torres Strait Islander people living with a disability due to the intersecting effects of discrimination and ableism.^5^ The *Royal Commission into Violence, Abuse, Neglect and Exploitation of People with Disability* revealed that Aboriginal and Torres Strait Islander people with disability face barriers to accessing the National Disability Insurance Scheme (NDIS) and disability services.^6^ A high proportion of Aboriginal and Torres Strait Islander people living with a disability experience racism, and those who experienced racism are more likely to avoid services.^7^ Aboriginal and Torres Strait Islander recipients of NDIS support are 28% less likely to receive care funded by the NDIS than non-Indigenous participants and report that some disability services are ‘unsafe, traumatising and inequitable’.^8^ Literature highlights the need to acknowledge the historical context of disability services in Australia because past injustices contribute to Aboriginal and Torres Strait Islander mistrust of government institutions and services.^9^,^12^

There are calls to decolonise disability literature and services, to understand better the perceptions and experiences of Aboriginal and Torres Strait Islander people living with disability, and to improve access to culturally appropriate, trauma-informed care.^5 6^ This paradigm shift would involve critically reflecting on the assumptions and narratives grounded in Western traditions and promoting Aboriginal and Torres Strait Islander leadership^13 14^ and knowledge systems.^15^ For example, the literature suggests that there is no word or translation for disability in Aboriginal and Torres Strait Islander languages and that the Western concept of disability—impaired bodily functioning that impacts one’s capacity to participate in society—is not compatible with Aboriginal and Torres Strait Islander peoples’ way of thinking^16^ and promotes individualistic binary hierarchies around abilities that are not consistent with their worldview.^17^ Rather, Aboriginal and Torres Strait Islander communities view impairments as characteristics of an individual’s lived experience and do not differentiate people with and without disability.^2 5 12 18^ Aboriginal and Torres Strait Islander people are more accepting of people living with disability,^9 19^ focusing on their strengths—attributes and capabilities to participate in various domains of life—rather than their deficits.^11^

The lack of disability support services for Aboriginal and Torres Strait Islander people in rural and remote Australia is well-documented.^2 20 21^ Western norms and concepts that have shaped this system—but do not reflect the values, practices and perspectives of Aboriginal and Torres Strait Islander peoples—have perpetuated the marginalisation of those living with disability and the barriers they face to access services.^16^ Although Aboriginal and Torres Strait Islander people perceive that the Western model of disability is culturally inappropriate, they must adopt this model to engage with support services.^9 18 21^ This need to adopt a worldview that does not align with their own has been shown to negatively affect Aboriginal and Torres Strait Islander people’s SEWB.^16 22^ A lack of understanding and adherence to Aboriginal and Torres Strait Islander cultural norms and practices among services (e.g., NDIS providers) may perpetuate confusion and mistrust towards support providers and highlights the need to strengthen the Aboriginal Community-Controlled sector^23^ and community connectors or navigators to co-deliver disability supports to Aboriginal and Torres Strait Islander communities.^24^ A recent review illustrated the need for further research to explore Aboriginal and Torres Strait Islander peoples’ experiences in NDIS planning in remote communities.^25^

Marninwarntikura Women’s Resource Centre (MWRC) is an Aboriginal Community-Controlled Organisation that services the Fitzroy Crossing and 32 surrounding Aboriginal communities in Western Australia. MWRC is internationally known for conducting research that captures and amplifies the values and preferences of Aboriginal communities in the Fitzroy Valley.^26^ In 2020, MWRC was engaged by the Australian National Disability Insurance Agency to consult community members with disability about the roll-out of the NDIS locally.^27^ At the time, MWRC and the University of Sydney were working on the Bigiswun Kid (Kimberley Kriol for adolescent) Project to better understand the needs of young people (17–20 years) in Fitzroy Valley.^28 29^ The NDIS consultation project identified facilitators and barriers that people with a disability experienced accessing services in the region, drawing on a multidimensional conceptualisation of access (time, space, price, quantity, quality, acceptability, information and awareness).^30^ It also helped to identify the facilitators and barriers young people experienced in accessing services. Approximately 20% of this cohort had a disability, which was diagnosed when the participants were children (aged 7–8 years); however, 10 years later, none were accessing support through the NDIS.^27^ A common barrier to accessing services was a difference in how disability is perceived and discussed by Aboriginal people in the Fitzroy Valley compared to staff from Western services. This barrier has received little research attention and is a crucial knowledge gap among government services. This study aims to understand this difference in worldviews of disability and identify strategies that can help bridge the gap between Aboriginal community members in the Fitzroy Valley and Western health and disability services.

## Methods

### Context

This project took place in the Fitzroy Valley (the Fitzroy Crossing town and 32 Aboriginal communities spread over a 400 km diameter), a very remote area in the Kimberley region of Western Australia.^31^ Approximately 4500 people live in the Fitzroy Valley, of whom 80% are Aboriginal and belong to five principal language groups: Bunuba, Gooniyandi, Walmajarri, Wangkatjungka and Nyikina. Kimberley Kriol is the primary language spoken in the region.^31^

### Design

Historically, Western research has perpetuated injustices against Aboriginal and Torres Strait Islander people,^32^,^35^ resulting in mistrust of researchers,^36^ a lack of Aboriginal and Torres Strait Islander voices in research^37^ and concern that research has not addressed the priorities of, or led to improvements in outcomes for, Aboriginal and Torres Strait Islander peoples,^38^,^40^ including in remote communities in the Kimberley region.^41^ These injustices have led to calls for decolonising Aboriginal and Torres Strait Islander health research and to centre the priorities and worldviews of non-Western peoples in knowledge generation and translation.^35^ The current study used an Aboriginal Participatory Action Research (APAR) approach, an Indigenous Research Method that centres Aboriginal researchers and communities in identifying research priorities and undertaking knowledge generation and dissemination in line with Aboriginal people’s preferences and ways of being and knowing.^42^ An Appreciative Inquiry Paradigm^43^ and research yarning approach^44 45^ were used to conduct interviews with Aboriginal community members. The interviews focused on strategies and solutions, rather than problems, aiming to generate collective solutions. The interviewer and community navigator took time to build and strengthen positive relationships with interviewees, and the interviewer memorised questions so they could be asked as part of a natural conversation. The topic of ‘perceptions of disability in the Fitzroy Valley compared with Western services’ was presented to the interviewees, who were then invited to yarn and share their views and experiences. The interviewee led the conversation, with the interviewer asking questions when it seemed appropriate. This approach was possible because the topics and questions were developed with the study’s Aboriginal authors following extensive discussions with community members. Drawing on the Aboriginal authors’ experience and mutual understanding of research ethics with Fitzroy Valley communities,^46 47^ this study was conducted following the National Health and Medical Research Council’s Ethical Conduct in Research with Aboriginal and Torres Strait Islander Peoples and Communities: Guidelines for Researchers and Stakeholders 2018^48^ and the Australian Institute of Aboriginal and Torres Strait Islander Studies Code of Ethics for Aboriginal and Torres Strait Islander Research.^49^ The study’s quality was assessed from an Aboriginal and Torres Strait Islander perspective using the Aboriginal and Torres Strait Islander quality appraisal tool (online supplemental material 1) and reported according to the consolidated criteria for reporting qualitative research checklist (online supplemental material 2).

### Sample and recruitment

We used purposeful sampling to recruit participants with lived experience or expertise on health or disability services in the Kimberley. This sample comprised two groups fulfilling the study’s inclusion criteria: (1) self-identified as an Aboriginal person from the Fitzroy Valley with lived experience of disability or supporting people with disability, or (2) lived and worked in the Kimberley for at least two years providing health or disability care to Aboriginal people. The study aimed to recruit an equal number of male and female participants. The authors developed a list of potential participants from their networks in the Kimberley region who they knew had extensive insight into these topics, who were then invited to join the study via email, phone or text. The study also used snowball sampling by inviting potential participants to suggest the names of other community members or colleagues who met the study’s inclusion criteria. Participants provided written informed consent. No potential participants refused to join the study, and no participants dropped out of the study.

### Data collection

Semi-structured interview guidelines were developed for data collection with Aboriginal community members (online supplemental material 3) and health and disability care professionals (online supplemental material 4). In late 2023, TS, LJR and EB conducted interviews with participants in person (n=15) or via video conference (Zoom and Microsoft Teams—health and disability professionals only) (n=4) at various locations throughout the Kimberley (workplaces, homes, community spaces). Interviews lasted for around 30–60 minutes and continued until information saturation was achieved, whereby subsequent interviews no longer yielded novel information or concepts. As Aboriginal researchers, EB and MB translated and provided contextual information. AP took notes during some interviews with health professionals. The interviews were recorded using an audio-recording device or video conference software, auto-transcribed and individually corrected by the researchers (TS, AP, HN). Identifiable information was anonymised to maintain the participants’ confidentiality and anonymity and sensitive information not pertinent to the study’s aims was removed. All data were securely stored and owned by the community and MWRC.

### Data analysis

Interview transcripts were uploaded to NVivo V.14 software and analysed using reflexive thematic analysis across six phases: (1) data familiarisation and note taking, (2) systematic coding, (3) generating initial themes from codes, (4) developing and revising themes, (5) defining themes and (6) write-up of results.^50^ TS and AP independently coded three transcripts in regular consultation with LJR. Field notes were consulted during this process to provide contextual insights and reflections, which enriched data interpretation. TS coded all the transcripts and developed a draft coding tree of overarching themes, sub-themes, representative quotes and interpretative notes. It was then discussed with AP and LJR, with their feedback integrated into a revised framework. The study’s preliminary themes were shared among coauthors (who included Aboriginal and non-Indigenous researchers) and community members (from, or with extensive experience living in, the Kimberley region or experience with Aboriginal family members with disability), who provided feedback, validation and contextualisation to ensure the findings aligned with the Aboriginal community’s perspectives and values. Since the Aboriginal researchers (MB, EB, EC and JD) and community members have the most profound understanding of the research topic, their views were given priority during these discussions. LJR and MB then co-designed a list of recommendations with Aboriginal community representatives who have experience with health and disability services to translate the research findings.

### Reflexive statement

This study employed a reflexive approach, acknowledging how the primary research team’s experiences and perspectives influenced the research process.^51^ This approach aimed to enhance the rigour and transparency of the research. TS and AP are both non-Indigenous Australians, with education and experience in public health research in cross-cultural settings, including the Kimberley region, and have direct or indirect lived experience with disabilities. Neither knew the participants before data collection. Through this study, they gained deeper insights into disability support services and diverse Aboriginal perceptions of disability. They reflected on how their evolving understandings shaped the data analysis and interpretations. LJR is a non-Indigenous research fellow who, at the time of the interviews, had lived and worked in the Fitzroy Valley for 4 years. During this time, she received formal cultural awareness training as well as ongoing daily cultural guidance and supervision from EC, EB, MB, JD and other senior Aboriginal community leaders. All from the Fitzroy Valley, EC is a Gooniyandi and Kija woman, JD is a Gooniyandi, Kija and Waanyi woman, EB is a Gooniyandi, Walmajarri and Ngarinyin woman and MB is a Bunuba man. EC, JD, ST, EJE, LJR, EB and MB had led several research projects in the Fitzroy Valley before this study, which focused on evaluating disability services and understanding the needs of young people with fetal alcohol spectrum disorder (FASD). EC, JD, LJR, EB and MB were known to all the Aboriginal community members interviewed, either personally or through previous projects.

### Patient and public involvement

Community members and MWRC identified the need for this research, supported the development of the research aims, assisted with participant recruitment, validation of results and co-design of recommendations. They will also be involved in the dissemination of the findings to communities, service providers and policy stakeholders.

## Results

### Participant demographics

A total of 19 participants were recruited: 7 Aboriginal community members (6 females and 1 male) and 12 service providers (10 females and 2 males). The Aboriginal community members were from seven communities in the Fitzroy Valley and all were primary carers of people with a disability. The people they cared for had a wide range of disabilities, such as intellectual disability, traumatic brain injury, FASD, autism spectrum disorder, loss of limbs, blindness, dementia, epilepsy and psychosocial disabilities (eg, severe schizophrenia). Four of the interviewees were senior Aboriginal leaders, and some were living with a disability. All had extensive experience accessing local health and disability services for themselves and their loved ones. Four of the seven had worked in the disability or health sector.

The health and disability professionals included senior leadership from health or disability services, clinicians and allied health professionals, such as speech pathologists, occupational therapists, psychologists and social workers. They each had experience working with people of all ages with a range of disabilities.

### Themes

Eight themes emerged from analysis of the interview transcripts. The community co-designed recommendations addressing these findings are presented in online supplemental material 6.

#### Aboriginal kinship system: a community strength and support for those living with disability

Both participant groups described the Aboriginal kinship system as a community strength and potential support for people living with disability, recalling examples when immediate and extended family or community members assisted others. Community participants described helping others as part of a broader ‘set of responsibilities’, explaining that the kinship system comes with a duty to one’s family and community. They recalled that it draws on different individuals based on their unique ‘strengths’, each playing ‘a different role with supporting’. These statements framed the kinship system as a implicit network of support, rather than an individual obligation to another person.

[Western services are] not understanding that the kinship systems that you’re born into service your community and it’s great, it’s inbuilt, it is like second nature to all of us (Community member)

Both groups recalled how the kinship system helped people living with disability to access support services. Community participants described the benefit of being present when a family member interacted with service providers, like a ‘doctor or a specialist’, supporting them emotionally or with communication—particularly if they required assistance with ‘high English’. A few community participants mentioned that support services often overlook the role of the kinship system in supporting those with disability:

They’re not recognising the work that people do and the relationships. They’re not giving value to the relationships and the kinship system of the people that are in the community that are in those positions … how vital they are to making things work and an easy transition for whoever is going through that system. (Community member)

Both groups discussed the importance of services integrating with the network strengths of the kinship system to support people living with a disability. Community members noted supporting caregivers would strengthen the kinship system and community as a whole around the person living with disability. However, both groups noted the complexity of integrating services with the kinship system due to community dynamics and the need to adhere to cultural practices and norms. Community members discussed Aboriginal staff or community navigators as key to addressing these complexities.

Strengthening the kinship system is key to strengthening communities (Community Member)

#### Aboriginal people in the Fitzroy Valley perceive disability as a social construct

In contrast to Western culture, Aboriginal people in the Fitzroy Valley perceive disability as a social construct. Community members recalled that, historically, disability was not a concept in Aboriginal culture and impairments were seen as unique characteristics like hair colour or height. Some service providers interpreted this worldview as the Aboriginal people’s acceptance of, or apathy towards, disability. Others hypothesed that this view was the reason why some Aboriginal parents did not seek a diagnosis or formal support for children with possible disabilities:

The contrast of people being so accepting to the point that the child might not have talked for three or four years, but they just accept it that’s what that kid is like … This contrast of just accepting everything and not questioning it and you just take that child for who they are, and I think that leads to not getting [an] early diagnosis because people just accept, and that’s what it’s like … I think they probably function at a level where … they’re just a little bit quirky, and because people are so accepting of quirky people. (Service provider)

Despite these different perspectives, both groups recalled that communities are accepting of, and great at normalising, disability. One service provider recalled that children with disability *“*were part of the community … It wasn’t a standout”. Others described how communities adjusted practices to ensure people living with a disability could participate:

Aboriginal people are very inclusive and very open-minded around disability … We need to change ourselves and our environment so that the person living with disabilities [can join in] … We don’t have to change them in order for them to fit in the environment … We need to change ourselves and the environment for that person to fit. (Community member)

#### Western medical model of disability differs from Aboriginal perceptions of disability in the Fitzroy Valley

Participants described how the Western medical model of disability differs from how disability is perceived in Aboriginal communities in the Fitzroy Valley. Community participants recalled that rather than perceiving disabilities as unique characteristics and focusing on an individual’s strengths and capabilities, the Western view isolates disability from the individual and concentrates on pathology or deficit:

With Aboriginal families, there’s a lot of strengths … With [the] Western world, when you do look at a disability … a lot of people look at the things that they can’t do. Whereas with Aboriginal people, it’s how do we sort of care for them and support them … We don’t look at the deficits; we look at the strengths … or the uniqueness in that person who has a disability. (Community member)

Community participants differed in their views on whether disability diagnoses are appropriate in Aboriginal communities. Some recalled that labels, such as ‘autism spectrum disorder’, were not congruent with how disabilities are spoken about. Others felt the diagnosis helped them understand their loved ones and that people in remote Aboriginal communities have the right to a diagnosis just like people in cities. These diverse opinions suggest that a one-size-fits-all approach towards diagnosis will not work. Some community member participants agreed that people with disabilities and their families should be given the option to explore a diagnosis if it is warranted, and if famlies are not interested in a diagnosis, the focus should be on their strengths and support needs. Both groups recalled that focusing on functionality and ‘sideways talking’ are culturally safe ways to discuss disabilities in the Fitzroy Valley. Sideways talking has two meanings, with the first relating to talking about an issue in an indirect or roundabout way:

Sideways talking is not being direct … You don’t talk right on the point because … You don’t want to blame … The sideways talking is where people can share their experiences … [It] gives them time to think, and that’s how people can help the next lot of people because that’s how we operate. For example, saying your brother has a mental illness, that’s direct … [Instead], sideways talking is when [you would say], he gets a bit stressed out every time this happens. (Community member)

The second relates to having a third person (a family member or trusted other) present during consultations, who can relay information between the person with disability and the service provider. Participants noted that both forms of sideways talking should be available to people with disabilities and their families when engaging with health and disability support services.

#### Aboriginal people from the Fitzroy Valley perceive different types of disabilities in various ways, which often depends on the visability of the disability (physical, cognitive, psychosocial)

Community members spoke about how they view different types of disabilities. Their perceptions of physical disabilities are shaped by the notion that these impairments are often visible, with a clear conceptual link between the condition and functionality. In contrast, less visible disabilities (such as cognitive or psychosocial disabilities) are more difficult to observe and, therefore, more complex to understand and distinguish in terms of how they impact function. Consequently, people living with physical disabilities are more likely to be identified and receive support than those with less visible disabilities:

When you look at the other disabilities like physical ones … they know straight away that person will need support. (Community member)

Both groups noted that a lack of awareness surrounding less visible disabilities contributed to misunderstandings around disability and limited access to support services. Some felt that community members misinterpreted behaviours associated with cognitive disabilities in children as misbehaviour or the result of poor parenting. They recommended that community and service provider education would help dispel these misconceptions surrounding less visible forms of disability and increase access to supports. Community members also identified a need for more education about the type of support services available for people with disability and people’s rights to access these (e.g., the NDIS and NDIS-funded services like allied health services and support in schools). This information would help empower people to actively seek the support they need. Community members also felt that those with lived experience of disability had the most credibility and trust to conduct education sessions:

[It] only takes one community leader, and the rest will follow … We need people who understand and talk about disability from our communities [to be] involved in the education around disability. If black fellas who feel comfortable talking about disability are there, they make other parents feel comfortable [to] open up and talk about disability. (Community member)

Both groups believed that cognitive disabilities were underdiagnosed in the Fitzroy Valley, and most knew multiple people who they thought might have an undiagnosed cognitive impairment. Some attributed this to the failure of schools to identify children with potential disabilities, long waitlists, limited access to assessments and a lack of culturally inappropriate assessments. Others felt that underdiagnosis was driven by healthcare providers’ avoidance and lack of knowledge, time and resources to identify cognitive disabilities. Service providers believed that underdiagnosis is driven by limited access to psychological or neuropsychological services in the Kimberley and a lack of culturally appropriate measures. They advocated for clinical psychologists and neuropsychologists to be embedded within mental health and allied health teams and supported by local Aboriginal Liaison Officers to ensure assessments are guided by cultural knowledge. A few participants from each group felt that some underdiagnosis was driven by feelings of guilt or fear related to FASD. However, others noted that this was partly driven by the increased awareness of FASD over other disabilities, like attention deficit hyperactivity disorder (ADHD).

A few participants from each group recalled that failure to recognise functional needs meant that many living with cognitive disabilities do not receive support, increasing the likelihood of secondary disabilities such as preventable illness (e.g., diabetes) or mental illness. They also highlighted the positive implications of identifying people living with disability, such as enhanced understanding of both the disability and the person’s support needs by their family, community members, schoolteachers and healthcare staff, as well as improved access to services.

Although service providers felt there was a lack of FASD awareness in some parts of the Kimberley, others noted that FASD awareness was high because of the community’s research and awareness raising. Community members spoke positively about the Fitzroy Valley’s actions to identify, address and prevent FASD. They also recalled how other disabilities may be mistaken for FASD because it is the only disability that has received attention in the Fitzroy Valley, suggested that more education is needed to address this misunderstanding and bring attention to all forms of disability. They also felt more education was needed to help people understand the functional needs—practical requirements and supports an individual needs to perform daily activities and participate in life—of people with FASD and how to support them.

I think we do have FASD days and stuff like that, but I think more information [is needed]. So, what Fitzroy does really well is the preventative stuff. So, we have implemented these community events, so we’re talking, we’re raising awareness and also prevention strategies around FASD, but I think supporting and having that education within family units around this child with FASD because every child with FASD is different. I think that’s where we need more work. (Community member)

One participant explained how understanding the functional needs of a person with FASD helped promote empathy among the family.

I think some of us had an understanding of it. Therefore, we were a lot more empathetic and supportive, and you know all of that. And then we had some that knew what the term was but didn’t actually know you know the full extent of their brain (Community member)

Service providers emphasised an association between FASD and trauma, with social inequality and inadequate living conditions contributing to disability and reduced outcomes for those living with disability. Community participants recalled some stigma associated with FASD, predominantly among children, which they felt could also be addressed with education about all disabilities. Both groups were concerned about mothers feeling guilt or blame and the stigma for people living with the diagnosis—since the term FASD focuses on the cause of the disability. While some felt increased access to education and services would help to reduce blame and stigma, a few suggested focusing on the function when speaking with families and providing an alternative term that people with FASD and their families can use following a diagnosis, like neurodevelopmental disorder.

Education is a good thing. Only takes one community leader and the rest will follow. The awareness has been raised, but not enough education. FASD has become a stigma for the community. More awareness of FASD everywhere and more education about what FASD means—education is our main thing, to get rid of the stigma rather than get rid of the disability. People think FASD only belongs to Fitzroy. They don’t realise it is all over the world. (Community member)

#### Focus should be on functional needs and support for people with disability

Participants from both groups suggested placing more emphasis on function, rather than a diagnostic label, when supporting people with disability and their families, particularly for FASD or possible FASD.

In the Western thing, of when you have to tick a box, that’s great to have your little label, but when you’re talking to the family … they need more help with this, they need time, there’s stuff to do with temper, all of that kind of stuff and how you can help you know [stay] cool, bring them back down, all of that kind of stuff, is where you talk about it at the family level yeah. (Community member)

One community member noted that even if a diagnosis, like autism spectrum disorder, is available, the family will often not use the label and instead talk about the person’s function (e.g., “that boy doesn’t talk”), because it is more tangible and less likely to offend. They also felt that more education about different diagnoses would make people more comfortable. One service provider offered a strength-based process she uses when supporting a family with a child with any developmental concerns, which considers the cultural context. The community members interviewed reviewed this process, agreeing and adding their insight (see online supplemental material 5).

#### Barriers to health and disability services

People living in remote Aboriginal communities face various barriers to accessing health and disability services. Both groups recalled how a lack of trust in Western service providers—resulting from trauma, historical marginalisation and oppression—is a barrier to accessing support. Participants felt that Aboriginal communities face a service barrier because they needed to adapt their practices to align with some Western systems and perceptions of disability, like talking about disability from a deficit-based, rather than a strengths-based, approach. Although some participants recalled the impact of geographic remoteness and harsh ‘environmental factors’ on access to services in the Kimberley, the main issue they raised was the high staff turnover and how it hinders relationships with service providers and continuity of care for families:

We have a very high turnover of staff, especially in the health sector … in all government departments actually … When you’re looking at, you know, building relationships and being routine and you know that regular person, I think for our young people, you know, and then all of a sudden, they up and leave (Community member)

Both groups stressed the importance of timely and adequate support following diagnosis. The complex NDIS process was often the reason for long waits between a diagnosis and accessing support:

The tricky thing is like giving someone a diagnosis and not having that follow-up … because there can be a really big gap between the time someone gets diagnosed … [and] the time they actually get on [the] NDIS and start seeing someone … It can be a really long time, especially out in communities where you’re doing outreach visits … I think diagnosis can be good, but it needs like immediate follow-up. (Service provider)

#### Decolonise disability services: align supports with Aboriginal ways of knowing, being and doing

Participants recommended decolonising disability services to ensure they align with Aboriginal ways of knowing, being and doing (see online supplemental material 6). Both groups recalled the importance of service providers building trust and relationships with communities through listening to, and showing respect for, their stories and experiences and maintaining continuity of relationships with families. Both groups also spoke about the need to deliver services in line with communities’ preferences, environments and ways of being—such as providing assessments and support in a safe and familiar location, often outside a clinic. Others highlighted the need to conduct services in a more flexible, slower process than what is expected in urban or Western settings:

If you’re coming from like a different setting, like a private practice … it’s like I need to get this done, this done, this done, an intervention plan and implement it, refine it, in this much time … just trying to go very, very fast. But it just won’t work … tone it back a bit. Just have conversations and listen … you’ve got to be listening (Service provider)

Both groups called for, and provided examples of, more culturally appropriate disability assessments and supports. These included using activities of daily living assessments relevant to remote Aboriginal communities or offering clients support that aligned with their everyday practices, like fishing or going out bush. They also recommended that more services adopt strengths-based approaches—focusing on their patients’ capabilities and unique qualities—when conducting assessments and delivering support. Participants also recommended increased cultural awareness and sensitivity training for service providers and the need to employ and train more community navigators—who provide knowledge and connection to communities beyond just language translation—to co-deliver services. However, due to confidentiality, both groups noted the need to work in teams so that clients could choose whether to speak with local Aboriginal staff known to them or non-Indigenous staff. Service provider participants described their positive experiences working with community navigators to deliver culturally appropriate services:

We’re really fortunate in some communities to have the community connectors … local people with knowledge of the local language and also some knowledge of the NDIS … I can’t stress it enough, though, how much you’re led by community and the people that are already working there or in that community connector role … someone that they can help translate or help with yeah that engagement because sometimes it’s engaged and sometimes it’s the language … multiple different things, but, yeah, generally have that … trusted person (Service provider)

## Discussion

This study explored the perceptions of disability among Aboriginal people in the Fitzroy Valley and Western health and disability service staff in the Kimberley region and co-designed recommendations to bridge the gap between the two groups. The findings highlight the need to decolonise health and disability services to align with Aboriginal knowledge and ways of being. To this end, it is essential to acknowledge and address the broader historical context of health and disability services in Australia—including the impacts of colonisation, trauma and racism on Aboriginal communities’ mistrust of Western services and their disempowerment in interactions with government services and institutions more broadly.^9^,^12^ Decolonisation of health and disability support services requires building trust and increasing access to culturally appropriate and strengths-based supports for Aboriginal and Torres Strait Islander peoples living with disability. This process must focus on community leadership and the involvement of community organisations.^13 14 16^ Our community co-designed, place-based recommendations (online supplemental material 6) outline various practical strategies to decolonise disability services. These include encouraging providers to focus on building trust with community members, adopting a flexible approach to align with the community members’ preferences, environment and ways of being, and offering more culturally appropriate assessments and supports. This research also highlighted the salient contribution of community navigators in assisting service providers in delivering disability support to remote Aboriginal communities in the Fitzroy Valley. As such, the authors recommend that all non-Indigenous health and disability services in the Kimberley, and similar remote settings, should be funded to employ Aboriginal community navigators. Funding is also needed to empower community members to complete the training required to become service providers, such as allied health assistant training, and provide more opportunities for people to access allied health tertiary education.

The study revealed that Aboriginal kinship systems are a community strength and may provide robust support for those living with disability in the remote Fitzroy Valley; however, they are not well understood by, or integrated with, Western service providers and systems. This finding aligns with previous evidence demonstrating the importance of immediate and extended family support in the care and well-being of Aboriginal and Torres Strait Islander children living with autism spectrum disorder and the expectation within communities that they must provide such support to their kin.^52^ Other research shows the significant role of community members in supporting Aboriginal and Torres Strait Islander people living with disability and that lack of support can result in caregiver hardship and burnout.^12^ The literature also demonstrates the economic and non-economic impacts of disability on caregivers in Aboriginal and Torres Strait Islander communities,^53^ which, in some cases, intersect with existing drivers of disadvantage, oppression,^54^ poverty and homelessness.^20^ Other research illustrates the challenges caregivers face in accessing support services for Aboriginal and Torres Strait Islander people living with a disability: delays in receiving assessments and support, difficulty navigating complicated government services^55^ and negative experiences of cultural stereotypes and racism perpetuated by service providers.^54^ Two recommendations arise from our findings and this literature. First, health and disability support services should aim to better understand and integrate with the strengths and resources of the Aboriginal and Torres Strait Islander kinship systems. Integrating with this diverse network of knowledge and expertise is likely to lead to better care and outcomes for those living with disability. Second, services must support caregivers (both financially and through capacity building) to sustainably and effectively identify and support those living with disability, thus reducing the likelihood of caregiver burnout or worsening inequality. Yet, while Aboriginal kinship systems are undeniably crucial in supporting Aboriginal people with disability, government services must acknowledge their own ongoing necessity by actively integrating with, rather than over-relying on, these vital community networks.

Our study shows that Aboriginal communities in the remote Fitzroy Valley perceive disability as a social construct and are accepting and inclusive of people living with a disability, often adjusting family or community practices to facilitate participation for those living with disability. This is consistent with past research,^5 18^ with Aboriginal and Torres Strait Islander peoples frequently perceive disability as ‘part of living’^9^ and not something that needs to be ‘cured’ or ‘fixed’.^19^ Further, communities often regard living with an impairment as disabling only when it impacts the person’s participation in individual, family and cultural domains.^5 18^ This notion is congruent with Australian research that highlights the benefit of supporting Aboriginal and Torres Strait Islander people living with disability to maintain community participation and connection to their land, family and cultures to lead fulfilling lives.^2^ Our current also study shows that Aboriginal communities in the Fitzroy Valley often view disability from a strength-based perspective, which sometimes creates challenges when engaging with the Western medical model that requires individuals to demonstrate evidence of their disability or health condition and its negative impacts to obtain a diagnosis, supports or treatment. This finding adds to the literature on the distinction, and potential tensions, between the Western and strengths-based Aboriginal views of disability.^11 16 17 22^ A lack of flexibility in service provision—including the time required to build relationships between service providers and Aboriginal participants and their families—is one way in which the current system fails to reflect the values, norms and practices of Aboriginal and Torres Strait Islander people.^21^ This literature highlights the need for Western services to adopt flexible, relationship-focused, strength-based approaches to engage Aboriginal and Torres Strait Islander communities more effectively and appropriately. However, additional funding for services is required to facilitate this shift in Aboriginal and Torres Strait Islander communities. Improved housing, training and support are also needed for service providers to address the high staff turnover rate in remote Aboriginal and Torres Strait Islander communities.

Our study revealed that remote Aboriginal communities in the Fitzroy Valley perceived types of disabilities differently, often recognising physical disabilities better than non-physical or ‘invisible’ disabilities. Disabilities that impact behaviour were more likely to be underdiagnosed and misunderstood, including by non-Indigenous staff working in the Fitzroy Valley. These findings are consistent with previous studies: some Aboriginal and Torres Strait Islander communities hold the notion that disability only constitutes physical or ‘visible’ impairments.^5 18^ As presented in the community co-designed recommendations, community members called for more education to raise awareness of the range of potential disabilities, available disability supports and services and rights of people with disability and their families.

As the location of Australia’s first population-based prevalence study of FASD^56 57^ and substantial community efforts to raise awareness about prenatal alcohol exposure and FASD,^26^ the Fitzroy Valley offers novel insights into the lived experiences following diagnosis and community recognition of FASD. The community members took pride in their town’s efforts to identify and support individuals with FASD. Although awareness was strong, community participants requested further education on understanding and supporting individuals living with FASD. Community members also felt that sometimes FASD was all people knew, and thus education and awareness raising needed to go beyond FASD to include education about the presentation and functional needs of a range of disabilities, particularly the less visible disabilities such as autism spectrum disorder, ADHD, acquired brain injury and psychosocial disabilities like schizophrenia. They also felt that increasing understanding of all disabilities would help reduce stigma, particularly among children. Other recommendations were to focus on function rather than the label, mainly when talking to families, and for health professionals to offer families an alternative term to FASD that they could use following a diagnosis, such as a neurodevelopmental disorder. One health service provider recalled a process they used to discuss function and support strategies with families that the community members reviewed, elaborated on and endorsed. These changes could help shift the current deficit-based view of FASD and other disabilities to a strengths-based view that aligns more with Aboriginal cultures, healing-informed approaches and holistic supports.^58^ Importantly, community members emphasised that Aboriginal people should have access to culturally safe, responsive services. As one community participants stated, “Aboriginal people have the right to access the same healthcare as anyone else in the country, including diagnostic services”—especially when a disability diagnosis is essential for accessing government services such as the NDIS, disability classroom support, carers allowance and the disability pension.

## Limitations

The findings should be considered alongside several limitations. First, the study used purposive sampling to recruit a small sample of participants, so the findings should not represent all service providers and Aboriginal community members in the Fitzroy Valley. However, the participants had direct experience with the research topic and the interviews reached saturation. While some of the themes arising from the analysis will be shared by other Aboriginal communities across the Kimberley and Australia, others may be unique to the Fitzroy Valley, particularly theme 5, as Fitzroy Valley communities have taken a unique approach to identify and respond to FASD. Second, some service provider interviews were conducted online, limiting the research team’s opportunity to build rapport with these participants and potentially restricting the depth of their expressed views and experiences. Third, service providers comprised most of the study’s sample; therefore, their views may be overrepresented in the findings. However, post-hoc Aboriginal community consultation on the results and community co-design of recommendations helped address this issue and centre community members’ insights. A strength of the study was that community member interviews were conducted alongside local Aboriginal community navigators and by a non-Indigenous researcher who had been living in the Fitzroy Valley for 4 years. This approach helped to build trust with this participant group and contextualise the discussions. Since the current study relied solely on qualitative data, future research could use valid, quantitative tools to measure Aboriginal people’s access to and satisfaction with disability services in Fitzroy Valley or other remote communities.

## Conclusion

Aboriginal people living with disability have unequal access to disability services, including the NDIS. Barriers include the ongoing impact of colonial history and injustices, which have contributed to mistrust in government institutions and services. Additionally, the Western medical model that underpins health and disability services often fails to align with Aboriginal people’s perceptions of disability and ways of being. Our findings highlight the need to decolonise disability services to provide equitable access to Aboriginal people living with disability. Community co-designed recommendations call for disability services to integrate with Aboriginal kinship systems, support caregivers, provide more flexible and culturally appropriate assessments and support, enhance cultural awareness and sensitivity training for service providers, adopte a strengths-based approach to disability and focus on functional needs and support strategies. Increased involvement of community navigators is salient to shift disability and other services to align with Aboriginal communities’ cultures, knowledge systems and ways of being.

## Supplementary material

10.1136/bmjopen-2024-093608 online supplemental file 1

10.1136/bmjopen-2024-093608 online supplemental file 2

10.1136/bmjopen-2024-093608 online supplemental file 3

10.1136/bmjopen-2024-093608 online supplemental file 4

10.1136/bmjopen-2024-093608 online supplemental file 5

10.1136/bmjopen-2024-093608 online supplemental file 6

## Data Availability

No data are available.
