# Supplementary material for: What are the Aboriginal worldviews of disability in the Fitzroy Valley? Aboriginal Participatory Action Research to develop strategies for decolonising disability services
Source: BMJ Open. 2025 Sep 1;15(9):e093608. doi: 10.1136/bmjopen-2024-093608 (PMC12406916; doi:10.1136/bmjopen-2024-093608)
Supplement: online supplemental file 1 [file bmjopen-15-9-s001.docx]

**Aboriginal and Torres Strait Islander Quality Appraisal Tool**

**Article citation:** What are the Aboriginal worldviews of disability in the Fitzroy Valley? Aboriginal Participatory Action Research to develop strategies for decolonising disability services

**Reviewer’s name:** Thomas Stubbs

**Date:** 02 July 2025

| **Question** | **Yes** | **Partially** | **No** | **Unsure** |
| --- | --- | --- | --- | --- |
| 1. Did the research respond to a need or priority determined by the community? | X |  |  |  |
| 2. Was community consultation and engagement appropriately inclusive? | X |  |  |  |
| 3. Did the research have Aboriginal and Torres Strait Islander research leadership? | X |  |  |  |
| 4. Did the research have Aboriginal and Torres Strait Islander governance? | X |  |  |  |
| 5. Were local community protocols respected and followed? | X |  |  |  |
| 6. Did the researchers negotiate agreements in regards to rights of access to Aboriginal and Torres Strait Islander peoples’ existing intellectual and cultural property? |  |  |  | X |
| 7. Did the researchers negotiate agreements to protect Aboriginal and Torres Strait Islander peoples' ownership of intellectual and cultural property created through the research? | X |  |  |  |
| 8. Did Aboriginal and Torres Strait Islander peoples and communities have control over the collection and management of research materials? | X |  |  |  |
| 9. Was the research guided by an Indigenous research paradigm? | X |  |  |  |
| 10. Does the research take a strengths-based approach, acknowledging and moving beyond practices that have harmed Aboriginal and Torres Strait peoples in the past? | X |  |  |  |
| 11. Did the researchers plan and translate the findings into sustainable changes in policy and/or practice? |  | X |  |  |
| 12. Did the research benefit the participants and Aboriginal and Torres Strait Islander communities? |  | X |  |  |
| 13. Did the research demonstrate capacity strengthening for Aboriginal and Torres Strait Islander individuals? |  | X |  |  |
| 14. Did everyone involved in the research have opportunities to learn from each other? | X |  |  |  |
