# Supplementary material for: What are the Aboriginal worldviews of disability in the Fitzroy Valley? Aboriginal Participatory Action Research to develop strategies for decolonising disability services
Source: BMJ Open. 2025 Sep 1;15(9):e093608. doi: 10.1136/bmjopen-2024-093608 (PMC12406916; doi:10.1136/bmjopen-2024-093608)
Supplement: online supplemental file 3 [file bmjopen-15-9-s003.docx]

**Understanding and Responding to Fetal Alcohol Spectrum Disorder in the Fitzroy Valley: Perceptions of community members and local service providers**

**Fitzroy Valley community members perception of FASD and other cognitive disabilities: Semi-structured Interview schedule**

**NOTE**: for the Interviewer: Proceed to ask questions in conversational style and probe for further thoughts where appropriate (e.g., “could you tell me more about …?” “What did you mean when you said …?”). The questions below will serve as a guide to the interview questions, which will be adapted as appropriate to individual participant circumstances**.**

**Disability**

1*.* Do you think that the way non-Aboriginal people in Australia think and talk about people with disability differs to how local people in the Fitzroy Valley think and talk about people with disability?

*(Only use example if needed) If you think about some people with disability that you know. Do their families talk about their loved ones with disability differently to how drive-in drive-out services do? Common language used by non-Aboriginal people, e.g., the NDIS is “this is Jo he has autism and an intellectual disability; he can’t speak and needs help with all aspects of his life”. Is that similar how families in the Valley would describe their children?*

2. If it is different, how is disability perceived or talked about in the Fitzroy Valley or can you give me some examples of how people would think or talk about someone with disability?

*(Only use this example if needed) For example, the literature suggests that there isn’t a word for disability in some Aboriginal languages and so disability isn’t seen as its own construct. Instead, the disability is part of the person, like his personality is part of him. So, talking about the disability is like talking about a personal part of the person. Do you think that is the same?*

- I’m going to tell you about some different types of disability and ask you to tell me; how they are perceived and talked about:
  - Physical (missing a leg, wheelchair, can’t see, can’t hear)
  - Cognitive (thinking, you explain something, but they can’t quite get it, or forget things a lot)
  - Mental health (schizophrenia, hearing thoughts, depression, sad and won’t leave the house; anxiety, you know those people that are so nervous that when someone knocks on the door, they run to you to open it or too afraid to do things they used to)

3. Within the Fitzroy Valley, are there some cultural norms or rules about speaking about other people that is maybe different to non-Aboriginal Australian culture?

*(Only use this example if needed) For example, is it ok to talk about other people? Would most parents be comfortable talking about their child’s struggles or challenges?)*

**Cognitive disability**

Now I’m going to describe some different types of cognitive impairments

1. Neurodevelopmental disorders (born with it like, autism, FASD)
2. Acquired brain injury (you weren’t born with it, but it happened later, like after a car accident, falling off a horse, or family violence incident where not just the outside of the head but the inside (brain) was injured)
3. Neurodegenerative (usually associated with older age, like dementia where the person’s memory is worse than that of other older people)

4. Do you think there are people in the Valley who might have these conditions but are unaware of or haven’t been assessed or diagnosed?

5. Can you think of one or two people, or quite a lot, like 5 or more?

6. Do you know anyone who has a cognitive impairment and has been assessed and diagnosed?

1. Do you know who identified that the person might need an assessment?
2. Do you know who did the assessment?
3. Were there any barriers to getting the assessments?
4. Was the diagnosis helpful? For example, to access services for the family?
5. Was there anything negative about getting the diagnosis?
6. Has the person been able to access any support or services as a result of the diagnosis?
7. Are there supports or services the person hasn’t been able to access?
8. Have you ever seen a report that was written after a cognitive assessment?
   1. If yes, were there things you like about the report
   2. Were there things you didn’t like about the report?

**FASD**

In a previous project, we spoke to parents of young people with FASD. Some parents talked about the difficulty they had with the diagnosis of FASD.

1. Being someone who knows the community well, can you think of reasons why people might have difficulty with the diagnosis? E.g., stigma, there is no help?
2. Can you think of how we could overcome these barriers? For example, awareness, education, additional support, or services.
3. Do you think there is good awareness about FASD in the Fitzroy Valley?
4. Do you hear people talking about FASD? If so, how do they talk about it?

**Shared language**

1. What are your thoughts on the term Fetal Alcohol Spectrum Disorder? Would you be comfortable saying you or your loved one has FASD, or would you prefer to use a different term, like neurodevelopmental disorder or on the spectrum?
2. What are your thoughts on the term disability?
3. Are there some words or ways you think health professionals and services should talk about people with disability?

**Workshop/resource content**

MWRC are planning to run workshops and develop resources for the Fitzroy Valley community members and health and disability professionals to share the information learned from this project. The goal is to foster a shared understanding of how disability is perceived among community members and non-Aboriginal health and disability services. We believe this will help bridge the gap and help increase access to services, which is particularly important with the benefits that can be had from the NDIS.

1. Is there anything you would like to learn or like your community to learn about cognitive disability or FASD?
2. Is there anything about the health or disability services that you think would be helpful for community members to better understand to support people, particularly people with disability?
3. Is there any information that you think would be helpful to include in the workshops/resources for:
   1. Health and disability professionals?
   2. Community members?
