# Supplementary material for: What are the Aboriginal worldviews of disability in the Fitzroy Valley? Aboriginal Participatory Action Research to develop strategies for decolonising disability services
Source: BMJ Open. 2025 Sep 1;15(9):e093608. doi: 10.1136/bmjopen-2024-093608 (PMC12406916; doi:10.1136/bmjopen-2024-093608)
Supplement: online supplemental file 4 [file bmjopen-15-9-s004.docx]

**Understanding disability in the Fitzroy Valley: Perceptions of community members and local service providers**

**Fitzroy Valley health professionals’ perception of FASD and other disabilities: Semi-structured Interview schedule**

**NOTE**: for the Interviewer: Proceed to ask questions in conversational style and probe for further thoughts where appropriate (e.g., “could you tell me more about …?” “What did you mean when you said …?”). The questions below will serve as a guide to the interview questions, which will be adapted as appropriate to individual participant circumstances.

**Disability**

1. Have you noticed differences in how disability more broadly is perceived or talked about by Aboriginal people in the Kimberley compared to non-Aboriginal people in urban places in Australia? If so, how?
2. What about specific types of disability
   1. Physical (missing a leg, wheelchair, can’t see, can’t hear)
   2. Cognitive (thinking, you explain something, but they can’t quite get it, or forget things a lot)
   3. Mental health (schizophrenia, hearing thoughts, depression)
3. If you travel across the Kimberley, have you noticed differences from one town or community to the next in how disability is perceived or discussed? If so, how?

**FASD**

1. Are you familiar with fetal alcohol spectrum disorder or FASD?
2. Do you think that health professionals in the Kimberley should be diagnosing people with FASD, why or why not?
3. Do you see people in your service who have a confirmed FASD diagnosis?
   1. Do they come with their birth mother or other carer (e.g. kinship care) or on their own?
   2. By whom are people being diagnosed (e.g., Department of Justice, Department of Communities, private services)?
   3. Is the diagnosis accessible in their health records (e.g., at the emergency department)?
   4. Do you find knowing the diagnosis helpful?
4. Do you think there is good awareness about FASD in the Fitzroy Valley?
5. Do you hear people talking about FASD? If so, how do they talk about it?

**Cognitive disability**

1. Do you see patients with other suspected cognitive impairment? If so, what kind(s)?
2. If you come across someone with a suspected cognitive impairment, what do you do? Referrals?
3. Have you played a role in diagnosing anyone with cognitive impairment? If so, what was your involvement?
4. Do you think cognitive impairment in the Kimberley is the same as, or more prevalent than, other regions (e.g., non-Aboriginal populations, in metro regions)?
5. Do you think cognitive impairments are being identified/managed as much here as other regions? If not, why?
6. If you are seeing people with potential cognitive disability who are not being formally identified, is this impacting their life in anyway? E.g., living your life with undiagnosed FASD, intellectual disability, traumatic brain injury, dementia (family, parenting, community, work, understand medical treatments/medications, pay bills, run household)
7. Do you feel there are any barriers to identifying possible cognitive impairment in this role/region? If so, what are you able to do to overcome/mitigate?
8. Are there any cultural factors that could contribute to difficulty identifying possible cognitive impairment?

**Shared language**

1. What are your thoughts on the term Fetal Alcohol Spectrum Disorder?
2. Are there some words or ways of talking about disability or FASD in the Kimberley that you have found helpful or unhelpful?

**Workshop/resource content**

MWRC are planning to run workshops and develop resources for the Fitzroy Valley community members and health and disability professionals to share the information learned from this project. The goal is to foster a shared understanding of how disability is perceived among community members and non-Aboriginal health and disability services. We believe this will help bridge the gap and increase access to services, which is particularly important given the benefits that can be gained from the NDIS.

1. Is there anything you would like to learn or want your staff to learn about cognitive disability or FASD?
2. Is there anything about the Fitzroy Valley community that you think would be helpful to understand better to help you support people, particularly people with disability?
3. Is there any information that you think would be helpful to include in the workshops/resources for:
   1. Health and disability professionals?
   2. Community members?
