# Supplementary material for: What are the Aboriginal worldviews of disability in the Fitzroy Valley? Aboriginal Participatory Action Research to develop strategies for decolonising disability services
Source: BMJ Open. 2025 Sep 1;15(9):e093608. doi: 10.1136/bmjopen-2024-093608 (PMC12406916; doi:10.1136/bmjopen-2024-093608)
Supplement: online supplemental file 5 [file bmjopen-15-9-s005.docx]

**Process for engaging and discussing function and support for Aboriginal people with disability or possible disability**

| **Step 1:**  Appreciate the importance of relationships to Aboriginal people and begin with rapport building, get to know the family, and allow the family to know a little about you to ensure a balanced connection. |
| --- |
| **Step 2:**  Focus on the person’s strengths, interests, and connection to kinship, culture, and country. This can include taking the time to understand a person’s place and role within the kinship system as a way of understanding their identity and mapping out a person’s supports within the kinship system. |
| **Step 3:**  Enquire about any concerns the family/person might have and explore these concerns, seek strategies or referrals – don’t just ask about concerns and then gloss over them. Responding with a plan of action will help build trust and rapport. |
| **Step 4:**  Discuss and explore ‘typical’ milestones for children or aspects of function for adults to allow people with disability or their family members to identify capabilities and unique traits/quirks/support needs. Once identified respond with a plan of action again, referrals and strategies. |
| **Step 5:**  Once strengths, capabilities and unique traits have been identified and strategies explored, possible diagnosis and/or the need for additional assessments could be explored with the family/person with disability, if appropriate to the family/person with disability. Most health professionals who follow this process find the discussion for diagnosis naturally arises with time – ideally prompted by the family. However, some community members did not like that doctors only discussed a diagnosis if asked as they felt it places a burden on families to know what to ask for. |
| Notes:  The person with disability and or their family should determine the pace of this process only moving to the next step when everyone is ready. Sometimes several sessions are required to build rapport which may seem time consuming than allowed in a Western medical model and has resource implications. Allowing the time to build trust will ensure a more productive and culturally appropriate service delivery model. However, increased funding for service providers is needed for them to facilitate this process in remote communities.  Either explicitly enquire/discuss the most appropriate terms/language used or be guided by the terms the family/community navigators use. Service providers could propose the following terms to discuss deficits: unique traits/unique characteristics/quirks/areas of support. Alternatively, focus on the strategies and paths people need to take to achieve a goal or address a deficit, as noted below in recommendation 5. |
