# Supplementary material for: What are the Aboriginal worldviews of disability in the Fitzroy Valley? Aboriginal Participatory Action Research to develop strategies for decolonising disability services
Source: BMJ Open. 2025 Sep 1;15(9):e093608. doi: 10.1136/bmjopen-2024-093608 (PMC12406916; doi:10.1136/bmjopen-2024-093608)
Supplement: online supplemental file 6 [file bmjopen-15-9-s006.docx]

**Recommendations for centring the Aboriginal worldview of disability in the Fitzroy Valley**

| **Theme 1: The Aboriginal kinship systems: a community strength and support for those living with disability** | **Recommendation 1:** Future resources and training should aim to increase service providers' understanding of the importance of kinship systems for Aboriginal people with disability and how it is structured and functions. This is to ensure that service providers know how to consider and discuss the kinship systems respectfully. |
| --- | --- |
|  | **Recommendation 2:** Health and disability service providers should enquire about a person’s kinship system. Taking the time to learn where a person fits within their kinship system will help understand the participant and their identity, what support they have available and what responsibilities/support they provide to their family. |
|  | **Recommendation 3:** When appropriate, the kinship system could be integrated into disability support (e.g., employing and training family to be allied health assistants). This should always be done by or with the involvement of Aboriginal staff. |
|  | **Recommendation 4:** Strategies should aim to strengthen and empower the kinship system and communities. e.g., by providing supports that ensure families who provide informal care can do so without burnout; by funding and upskilling champions in each Aboriginal community to understand the Western way of the NDIS and disability so that they can support people in their community to navigate the health and disability services; by upskilling community members to be allied health assistants who help ensure people can implement strategies learned through formal sessions with health professionals; and provide more opportunities for people to access tertiary education to become allied health and health professionals. |
| **Theme 2: Aboriginal people in the Fitzroy Valley perceive disability as a social construct** | **Recommendation 5:** Service providers need to understand the historical and current Aboriginal worldview of disability and learn how to perceive and discuss disability as a social construct. This can be achieved by reframing discussions so that greater emphasis is placed on how environments can support a person with a disability rather than on how that person needs to change/learn/develop. For example, when discussing allied health support, such as speech therapy, providers should focus on how the family can incorporate strategies to promote language development rather than focusing on what a person cannot do. This difference can be subtle and take time for professionals to retrain the way they talk but doing so will have a big impact on how they are perceived by people with disability and their family. |
| **Theme 3: Western medical model of disability differs from Aboriginal people’s perceptions of disability in the Fitzroy Valley** | **Recommendation 6:** Service providers must understand that the Western medical model of disability differs from how disability is perceived and discussed in the Fitzroy Valley. Instead of focusing on deficits, focus on capabilities, strengths and function, and how to support/care for a person. When appropriate, use sideways talking (either using a third person to help have the discussion or talking in a roundabout way).  When working with Aboriginal people in the Fitzroy Valley, service providers should use the process for discussing function and support for Aboriginal people with disability outlined in Table 1. |
| **Theme 4: Aboriginal people perceive different types of disabilities in various ways, often depending on how visible they are (physical, cognitive, psychosocial)** | **Recommendation 7:** Raising community and service provider education would help dispel misconceptions surrounding less visible forms of disabilities (autism spectrum disorder, intellectual disability, acquired brain injury) and increase access to support services. Education for community members should also include information about disability supports, such as the NDIS, educational supports, and the types of services available to people with disability (allied health therapies, respite care) as well as the rights of people with disability and their families.  Strategies for educating communities:   - Education in communities should be led by Aboriginal people with lived experience with disability in collaboration with health professionals. - Workshops should be implemented in collaboration with the community, guided by leaders/elders/people with experience or passion for disability to decide how their community should be informed. - Ensure training is responsive so the training touches on issues important to each community. However, it is also essential that the training is consistent across communities; everyone is given the same core information so a person with a disability can be understood and supported when they go from one community to another. Non-Indigenous trainers must come across clearly, be mindful of language differences and guided by local Aboriginal trainers. Use visuals and be interactive; the learning must happen both ways (e.g., in ‘two-way teaching’ the group teaches the trainers who in return teach the group). - Identifying and supporting people in each community to become ‘champions’ who can be upskilled to support their families to understand and navigate disability and related services would help empower local community members and ensure capacity building continues and is maintained. |
|  | **Recommendation 8:** Employing Clinical psychologists or neuropsychologists embedded in local mental health or allied health teams and supported by local Aboriginal Liaison Officers would increase access to diagnostic services and ensure assessments are guided by place-based cultural knowledge. |
| **Theme 5: There is good awareness of FASD in the Fitzroy Valley. The community members would like more education about the functional needs of people with FASD and how to support them.** | **Recommendation 9:** Both community members and service providers would like more training on the functional needs of people with FASD and strategies to support them. However, this training should be provided alongside training in other forms of disability so that FASD is not the only disability people know and understand. |
|  | **Recommendation 10:** Health professionals could provide families with an alternative term for FASD that they can use if it makes them more comfortable (e.g., neurodevelopmental disorder). |
|  | **Recommendation 11:** Some participants felt that not recognising the functional needs associated with FASD increased the risk of secondary disabilities in later life, so access to diagnostic/functional capacity assessment services is crucial. |
| **Theme 8: Decolonise disability services: align support services with Aboriginal ways of knowing, being and doing** | **Recommendation 12:** Ways to help ‘decolonise’ disability services:   - Service providers need to take the time to build trust and relationships by listening to and showing respect for community members' stories and experiences and maintaining the continuity of relationships with families. - Service providers should appreciate that the mistrust Aboriginal people often have with Western services may be due to the continued impacts of colonisation, limited funding of services, and high staff turnover. Services should ensure that providers are reliable and consistent to help earn trust. - Services should be delivered according to communities' preferences, environments, and ways of being, for example, providing support in a safe and familiar setting and ensuring the appropriate process and permission is followed before entering a community. - Services must be more flexible and adapt the pace to work with the family, e.g., provide more time for rapport building. Yet, additional funding is required to facilitate services providers to adopt this approach. - Services should offer more culturally appropriate assessments and supports, e.g., using activities of daily living assessments relevant to remote Aboriginal communities and offering support alongside their everyday practices, like fishing or going out bush. - Services should be mindful of the need to adopt a strength-based approach, focusing on the individual’s capabilities and unique qualities. - Increased cultural awareness and sensitivity training is required for service providers. - More community navigators should be employed and trained across all health and disability services. The community members interviewed strongly endorsed this recommendation. Community navigators will help ensure that all the above points are implemented. - Service providers should work in teams so participants/patients can choose to talk to someone that they do or do not know. |
