# Supplementary material for: What are the Aboriginal worldviews of disability in the Fitzroy Valley? Aboriginal Participatory Action Research to develop strategies for decolonising disability services
Source: BMJ Open. 2025 Sep 1;15(9):e093608. doi: 10.1136/bmjopen-2024-093608 (PMC12406916; doi:10.1136/bmjopen-2024-093608)
Supplement: Supplementary file 7 [file bmjopen-2024-093608.reviewer_comments.pdf]

PEER REVIEW HISTORY

BMJ Open publishes all reviews undertaken for accepted manuscripts. Reviewers are asked to complete a checklist review form and are provided with free text boxes to elaborate on their assessment. These free text comments are reproduced below.

ARTICLE DETAILS

Title (Provisional)

What are the Aboriginal worldviews of disability in the Fitzroy Valley? Aboriginal Participatory Action Research to develop strategies for decolonising disability services

Authors

Stubbs, Thomas; Bedford, Mudge; Bear, Emma; Carter, Emily; Pickard, Anita; Davies, Jadnah; Thomas, Sue; Martiniuk, Alexandra LC; Elliott, EJ; Rice, Lauren J

VERSION 1 - REVIEW

**Reviewer**	**1**
**Name**	**Badry, DE**
**Affiliation**	**University of Calgary, Faculty of Social Work**
**Date**	**11-Dec-2024**
**COI**	**None**

Thank you for this interesting paper that clearly articulates the unique response to disability in the Fitzroy Valley. The paper is strong and highlights critical disparities in terms of disability supports and lack of access to NDIS. As you identified 28% of Indigenous participants are less likely to access NDIS funding. Data collection well explained. Supplementary material is helpful. One comment on supplementary material 4 (word document) - FASD - 3c. Is the diagnosis accessible in their health records (e.g., ED) - can you ED in full as it is unknown what this means.

The presentation of sideways talking as a conversational strategy to address issues of disability in a respectful way was very interesting and well stated demonstrating the strength of community.

On p. 15 l. 37 - Increase the likelihood of secondary disabilities such as preventable illness – note - incarceration is not a secondary disability so I would recommend this be removed from the list – citation required for secondary disability reference - possibly Streissguth.

On p. 16- l. 47– a few suggested focusing on the function - Expand on the meaning of function a bit for readers to understand this better – needs some clarity. I.e. focus on how

the individual with FASD functions social/emotionally or functions generally, or as noted in theme 6 where you focus on functional needs as noted. This is not major but could help clarify the meaning of function to the reader.

p. 17 l. 10 more emphasis on function - Again expand on the meaning of function briefly for clarity - your later explanation such as "the kid doesn't talk" was very clear but comes later in the paper.

p. 18 . 10-11 – suggest responding with a 'plan' of action

l 18 respond with action 'plan'

l. Step 5 – l. 25 suggestion - or use suggestion below -family/person with disability if this is considered helpful to the family/person with disability

if appropriate, - put a comma after appropriate

Thank you for the insights which truly illuminate the challenges faced in accessing disability supports for the commmunity. p. 25 - While Aboriginal kinship supports are a great source of strength and provide much needed supports to individuals and families, they also carry the responsibility in the absence of available disability supports that could offer funding and service provision. Disability has been reframed as a critical construct that is accepted within the Fitzroy Valley by kin who care for and share responsibility for family and relatives with disability. This comes naturally but the system that should support persons with disabilities also needs to be at the table supporting equity and the rights of citizens with disabilities to access disability funding and support. A human rights lens could be helpful to briefly in your discussion.

Table 1 strongly articulates the process. I offer the following suggestion for your Table heading.

Table 1 excellent information on the process of engagement with the community - suggesting you emphasize your process of engagement which was so reflective in your outcomes yielding a depth understanding of what is involved in supporting community in accessing disability supports. Table 2 also provides excellent recommendations.

p. 17 l. 46-47 Process of 'engagement' for discussing function and support for Aboriginal people with disability or suspected disability

The term possible could replace suspected - that is what CanFASD is currently using instead of suspected FASD, particularly without diagnosis. See the document on Common Messages and FASD - https://canfasd.ca/wp-content/uploads/publications/CanFASD-Common-Messages-Guide-2023_FINAL-1.pdf

Thank you for this paper which offers critical insights to add to the discourse on disabilities, particularly in the Fitzroy Valley, and access or lack of access to disability supports which rightfully belong to all citizens with disabilities. Your discussion on decolonizing disability is essential and necessary. The work of Lavern & Locust (2013) also offered profound insights

into the Indigenous worldview and disability Native American Communities on Health and Disability: A Borderland Dialogues (March 2013). DOI: 10.1057/9781137312020. I see you referenced Lovern. Thank you for the opportunity to review this paper.

ISBN: 978-1-349-45606-2

**Reviewer**	**2**
**Name**	**Loyola-Sanchez, Adalberto**
**Affiliation**	**University of Alberta Faculty of Medicine and Dentistry**
**Date**	**01-May-2025**
**COI**	**None**

This qualitative research study aimed to understand the worldview differences regarding disability between Aboriginal community members and Western Society in the Fitzroy Valley in Australia. An important strength of this study is that is a report of data owned by the Indigenous communities involved. The authors also evaluated the quality of the participatory action research conducted according to the Aboriginal and Torres Strait Islander values and views. Overall, this is a very important qualitative work that provides concrete recommendations to improve the services provided to Indigenous people living with disabilities in the Fitzroy Valley, Australia, derived from a robust participatory action research strategy.

Following, I provide some recommendations to increase the quality of the report based on my review following the COREQ guidelines:

Researcher's reflexivity- I recommend to authors to add a description authors' pre study pre conceptions, reasons and interests for doing the study along with a description of what participants knew about the researchers at the moment of the interviews. This is part of the report for a reflexive thematic analysis. This could be done as a subsection in the methods section, which will facilitate reading.

Sample and recruitment- I recommend to further describe which characteristics were purposively looked for during the sample strategy, for example gender, type of disability, type of professional, etc

Data collection- I recommend authors briefly state if there were potential participants who refused or dropped out of the study and if so to reflect on how their "silences" could have affected the results.

Data collection- I recommend to authors to further describe what is meant by "various locations" when referring to setting of data collection. Do these locations include people's homes, working settings, community spaces, etc?

Results- I recommend to provide a description of the important characteristics of participants, such as age, gender, type of disability, profession. This could be done in the form of a table.

Analysis- I recommend to authors to describe how field notes were integrated to the analysis.

Data collection- I recommend authors to describe what type of data saturation assessment they followed to decide when to stop data collection.

Data analysis- I recommend to authors to provide a description of the coding tree that led to the generation of main themes.

Results- I recommend to provide an example of how "sideways talking" looks like. This is an important finding and I am not sure that non Aboriginal readers will know exactly what this means.

Section/Topic	Item No	Checklist item	Reported on page No
**Domain 1: Research team and reflexivity**			
Personal Characteristics			
Interviewer/facilitator	1	Which author/s conducted the interview or focus group?Interviewer/facilitator	Two aboriginal researchers and one non aboriginal researcher conducted the interviews.
Credentials	2	What were the researcher's credentials? E.g. PhD, MD	
Occupation	3	What was their occupation at the time of the study?	
Gender	4	Was the researcher male or female?	
Experience and training	5	What experience or training did the researcher have? Relationship with participants	
Relationship with participants			
Relationship established	6	Was a relationship established prior to study commencement?	Time allocated to establish relationship between interviewers and participants.
Participant knowledge of the interviewer	7	What did the participants know about the researcher? e.g. personal goals, reasons for doing the research	Researcher's reflexivity- I recommend to authors to add a description authors' pre

			study pre conceptions, reasons and interests for doing the study along with a description of what participants knew about the researchers at the moment of the interviews. This is part of the report for a reflexive thematic analysis. This could be done as a subsection in the methods section, which will facilitate reading.
Interviewer characteristics	8	What characteristics were reported about the interviewer/facilitator? e.g. Bias, assumptions, reasons and interests in the research topic	
**Domain 2: study design**			
Theoretical framework			
Methodological orientation and Theory	9	What methodological orientation was stated to underpin the study? e.g. grounded theory, discourse analysis, ethnography, phenomenology, content analysis	The authors followed an Aboriginal Participatory Action Research approach with an appreciative inquiry paradigm and a research yarning approach. This approach followed a pragmatic worldview focused on

			generating collective solutions to specific problem. The authors followed a thorough non-colonial approach to research.
Participant selection			
Sampling	10	How were participants selected? e.g. purposive, convenience, consecutive, snowball	Sample and recruitment- I recommend to further describe which characteristics were purposively looked for during the sample strategy, for example gender, type of disability, type of professional, etc.
Method of approach	11	How were participants approached? e.g. face-to-face, telephone, mail, email	e-mail, phone or text.
Sample size	12	How many participants were in the study?	19, 7 Indigenous persons with disability and 12 professionals.
Non-participation	13	How many people refused to participate or dropped out? Reasons?	Data collection- I recommend authors briefly state if there were potential participants who refused or dropped out of the study and if so to reflect

			on how their "silences" could have affected the results.
Setting of data collection	14	Where was the data collected? e.g. home, clinic, workplace	Data collection- I recommend to authors to further describe what is meant by "various locations" when referring to setting of data collection. Do these locations include people's homes, working settings, community spaces, etc?
Presence of non-participants	15	Was anyone else present besides the participants and researchers?	Not relevant
Description of sample	16	What are the important characteristics of the sample? e.g. demographic data, date	Results- I recommend to provide a description of the important characteristics of participants, such as age, gender, type of disability, profession. This could be done in the form of a table.
Data collection			
Interview guide	17	Were questions, prompts, guides provided by the authors? Was it pilot tested?	Described in supplementary material.
Repeat interviews	18	Were repeat interviews carried out? If yes, how many?	No
Audio/visual recording	19	Did the research use audio or visual recording to collect the data?	Audio recorded and

			auto transcribed.
Field notes	20	Were field notes made during and/or after the interview or focus group?	Analysis- I recommend to authors to describe how field notes were integrated to the analysis.
Duration	21	What was the duration of the interviews or focus group?	30 to 60 minutes
Data saturation	22	Was data saturation discussed?	Data collection- I recommend authors to describe what type of data saturation assessment they followed to decide when to stop data collection.
Transcripts returned	23	Were transcripts returned to participants for comment and/or correction?	
Domain 3: analysis and findings<br>Data analysis			
Number of data coders	24	How many data coders coded the data?	Reflexive thematic analysis by three researchers.
Description of the coding tree	25	Did authors provide a description of the coding tree?	Data analysis- I recommend to authors to provide a description of the coding tree that led to the generation of main themes.
Derivation of themes	26	Were themes identified in advance or derived from the data?	Themes were identified from data.
Software	27	What software, if applicable, was used to manage the data?	Nvivo

Participant checking	28	Did participants provide feedback on the findings?	There was a strong community engagement process that assured community feedback on coding and interpretations.
Reporting			
Quotations presented	29	Were participant quotations presented to illustrate the themes / findings? Was each quotation identified? e.g. participant number	Yes, themes contain quotes all logical and coherent in terms of themes.
Data and findings consistent	30	Was there consistency between the data presented and the findings?	Yes, interpretations logically followed from results (themes).
Clarity of major themes	31	Were major themes clearly presented in the findings?	Yes<br><br>Results- I recommend to provide an example of how "sideways talking" looks like. This is an important finding and I am not sure that non Aboriginal readers will know exactly what this means.
Clarity of minor themes	32	Is there a description of diverse cases or discussion of minor themes?	Not applicable

This qualitative research study aimed to understand the worldview differences regarding disability between Aboriginal community members and Western Society in the Fitzroy Valley in Australia. An important strength of this study is that is a report of data owned by the Indigenous communities involved. The authors also evaluated the quality of the

participatory action research conducted according to the Aboriginal and Torres Strait Islander values and views. Overall, this is a very important qualitative work that provides concrete recommendations to improve the services provided to Indigenous people living with disabilities in the Fitzroy Valley, Australia, derived from a robust participatory action research strategy.

VERSION 1 - AUTHOR RESPONSE

**Reviewer 1's Comments to the Author**	
Thank you for this interesting paper that clearly articulates the unique response to disability in the Fitzroy Valley. The paper is strong and highlights critical disparities in terms of disability supports and lack of access to NDIS. As you identified 28% of Indigenous participants are less likely to access NDIS funding. Data collection well explained. Supplementary material is helpful.	Thank you for your encouraging feedback.
One comment on supplementary material 4 (word document) - FASD - 3c. Is the diagnosis accessible in their health records (e.g., ED) - can you ED in full as it is unknown what this means.	We have revised both interview schedules (supplementary materials 3 and 4) to spell out acronyms and ensure they are understood by a wider audience.
The presentation of sideways talking as a conversational strategy to address issues of disability in a respectful way was very interesting and well stated demonstrating the strength of community.	Thank you for your encouraging feedback.
On p. 15 l. 37 - Increase the likelihood of secondary disabilities such as preventable illness – note - incarceration is not a secondary disability so I would recommend this be removed from the list – citation required for secondary disability reference - possibly Streissguth.	"incarceration" removed from sentence – see page 15.<br><br>We also removed mention of incarceration as secondary disability – see Table 2, page 22.<br><br>Thank you kindly for recommendation; however, citation in results were not included.
On p. 16- l. 47– a few suggested focusing on the function - Expand on the meaning of function a bit for readers to understand this better – needs some clarity. I.e. focus on how the individual with FASD functions social/emotionally or functions generally, or as noted in theme 6 where you focus on functional needs as noted. This is not major but could help clarify the meaning of function to the reader. p. 17 l. 10 more emphasis on function - Again expand on the meaning of function briefly for clarity - your later explanation such as "the kid doesn't talk" was very clear but comes later in the paper.	So readers can better understand, Theme 5 now starts with our description of functional needs: "the practical requirements and supports an individual needs to perform daily activities and participate in life – see page 14.
p. 18 . 10-11 – suggest responding with a 'plan' of action l 18 respond with action 'plan'	As recommended, we have revised text to "plan of action" in two sections – see Table 1, page 17.

l. Step 5 – l. 25 suggestion - or use suggestion below - family/person with disability if this is considered helpful to the family/person with disability<br>if appropriate, - put a comma after appropriate	We have added a comma after the word appropriate – see Table 1, page 17.
Thank you for the insights which truly illuminate the challenges faced in accessing disability supports for the commmunity. p. 25 - While Aboriginal kinship supports are a great source of strength and provide much needed supports to individuals and families, they also carry the responsibility in the absence of available disability supports that could offer funding and service provision. Disability has been reframed as a critical construct that is accepted within the Fitzroy Valley by kin who care for and share responsibility for family and relatives with disability. This comes naturally but the system that should support persons with disabilities also needs to be at the table supporting equity and the rights of citizens with disabilities to access disability funding and support. A human rights lens could be helpful to briefly in your discussion.	We thank you for this helpful feedback. As suggested, we have added the following text to the end of this paragraph: "Yet, while Aboriginal kinship systems are undeniably crucial in supporting Aboriginal people with disability, government services must acknowledge their ongoing necessity by actively integrating with, rather than over-relying on, these vital community networks."<br><br>Please see page 33.
Table 1 strongly articulates the process. I offer the following suggestion for your Table heading.<br>Table 1 excellent information on the process of engagement with the community - suggesting you emphasize your process of engagement which was so reflective in your outcomes yielding a depth understanding of what is involved in supporting community in accessing disability supports. Table 2 also provides excellent recommendations.	As suggested, we have added "engaging" to the title of the table – please see Table 1, page 16
p. 17 l. 46-47 Process of 'engagement' for discussing function and support for Aboriginal people with disability or suspected disability<br>The term possible could replace suspected - that is what CanFASD is currently using instead of suspected FASD, particularly without diagnosis. See the document on Common Messages and FASD - https://canfasd.ca/wp-content/uploads/publications/CanFASD-Common-Messages-Guide-2023_FINAL-1.pdf	As suggested, we have replaced the term "suspected" with "possible" – see Table 1, page 16.<br><br>We have also replaced "suspected" with "possible" in the preceding paragraph – page 16.
Thank you for this paper which offers critical insights to add to the discourse on disabilities, particularly in the Fitzroy Valley, and access or lack of access to disability supports which rightfully belong to all citizens with disabilities. Your discussion on decolonizing disability is essential and necessary. The work of Lavern & Locust (2013) also offered profound insights into the Indigenous worldview and disability Native American Communities on Health and Disability: A Borderland Dialogues (March 2013). DOI: 10.1057/9781137312020. I see you referenced	Thank you for your encouraging feedback.

Lovern. Thank you for the opportunity to review this paper. ISBN: 978-1-349-45606-2	
**Reviewer 2's Comments to the Author**	
This qualitative research study aimed to understand the worldview differences regarding disability between Aboriginal community members and Western Society in the Fitzroy Valley in Australia. An important strength of this study is that is a report of data owned by the Indigenous communities involved. The authors also evaluated the quality of the participatory action research conducted according to the Aboriginal and Torres Strait Islander values and views. Overall, this is a very important qualitative work that provides concrete recommendations to improve the services provided to Indigenous people living with disabilities in the Fitzroy Valley, Australia, derived from a robust participatory action research strategy. Following, I provide some recommendations to increase the quality of the report based on my review following the COREQ guidelines (see document attached):	Thank you for your encouraging feedback.
Researcher's reflexivity- I recommend to authors to add a description authors' pre study pre conceptions, reasons and interests for doing the study along with a description of what participants knew about the researchers at the moment of the interviews. This is part of the report for a reflexive thematic analysis. This could be done as a subsection in the methods section, which will facilitate reading.	We have added a "Reflexive Statement" sub-heading to the methods section. It outlines the researchers' backgrounds, preconceptions and factors influencing the study – see page 9. We also added a sentence to the above section noting how the views of the Aboriginal researchers were privileged.
Sample and recruitment- I recommend to further describe which characteristics were purposively looked for during the sample strategy, for example gender, type of disability, type of professional, etc	We added details on the participant characteristics as well as a sentence to state that the study aimed to recruit a near-equal number of male and female participants - see page 8
Data collection- I recommend authors briefly state if there were potential participants who refused or dropped out of the study and if so to reflect on how their "silences" could have affected the results.	No potential participants refused to join the study, and no participants dropped out of the study. We have added this text to the bottom of the Sample and Recruitment sub-heading – see page 8
Data collection- I recommend to authors to further describe what is meant by "various locations" when referring to setting of data collection. Do these locations include people's homes, working settings, community spaces, etc?	We've clarified "various locations" on page 8 to specifically mean "workplaces, homes, and community spaces."
Results- I recommend to provide a description of the important characteristics of participants, such as age, gender, type of disability, profession. This could be done in the form of a table.	We added a section at the start of the results outlining the participant demographics. We felt it was best to describe examples rather than outline specific numbers in a Table as the latter will likely identify people.

Analysis- I recommend to authors to describe how field notes were integrated to the analysis.	We have added details on how the field notes were integrated into the analysis – see Data Analysis, page 9
Data collection- I recommend authors to describe what type of data saturation assessment they followed to decide when to stop data collection.	We have added details on how the study used information saturation – see Data Collection, middle paragraph, page 8-9
Data analysis- I recommend to authors to provide a description of the coding tree that led to the generation of main themes.	We have added further details on development of the coding tree, including overarching themes, sub-themes, representative quotes, and researcher interpretative notes – see Data analysis, middle of paragraph, page 9
Results- I recommend to provide an example of how “sideways talking” looks like. This is an important finding and I am not sure that non Aboriginal readers will know exactly what this means.	We have updated the text slightly to highlight the two examples of sideways talking, as recalled from community member participants. Further, we added some further quotes to provide additional examples of sideways talking. We hope this revised text helps non-Aboriginal readers understand sideways talking – see page 13

VERSION 2 - REVIEW

Reviewer 1

Name Badry, DE

Affiliation University of Calgary, Faculty of Social Work

Date 17-Jul-2025

COI

Thanks you for your detailed attention to the feedback and completion of the required revisions. I have reviewed all the requested revisions and they are attended to in a satisfactory manner. As noted in my earlier review, this article offers important insights into the experiences of people in the Fitzroy Valley in relation to the Aboriginal Worldview of Disability.
